# Central Nervous System Progression in Folliculotropic Mycosis Fungoides

**DOI:** 10.1155/crh/6972475

**Published:** 2025-12-22

**Authors:** Cher Ying Foo, Aditya Sanjeevi, Harkarandeep Singh

**Affiliations:** ^1^ Department of Internal Medicine, Rochester General Hospital, Rochester, New York, USA, rochesterregional.org

## Abstract

**Background:**

Central nervous system (CNS) involvement by cutaneous T‐cell lymphomas (CTCL) is exceptionally rare and is associated with a poor prognosis. Folliculotropic mycosis fungoides (FMF) is a rare subtype of CTCL and often has more aggressive clinical behavior compared to classic MF. The risk factors for CNS progression in patients with primary CTCL are not well understood.

**Case Summary:**

We present the case of a 75‐year‐old male with a history of Stage IA FMF, who developed progressive neurologic and functional decline over six months. Workup revealed marked eosinophilia, and CSF analysis showed monoclonal T‐cell population with positive T‐cell receptor (TCR) gene rearrangement. Flow cytometry further showed an atypical CD4+ T‐cell population with loss of CD7, consistent with the immunophenotype of patient’s cutaneous MF, supporting secondary CNS involvement. An MRI of the brain showed T2 hyperintensity in the pons and right middle cerebellar peduncle, with low signal intensity throughout the bone marrow of the skull, concerning for CNS progression of FMF. The patient was started on high‐dose steroids, which led to improvement in eosinophilia. Due to transaminitis, high‐dose methotrexate therapy was deferred, and the patient was initiated on intrathecal (IT) chemotherapy with methotrexate and cytarabine. Following five cycles of IT chemotherapy, the patient’s neurologic status significantly improved, and CSF analysis showed resolution of the atypical T‐cell population.

**Conclusion:**

This case highlights the rare occurrence of CNS progression of FMF, even in the absence of lymphatic involvement and with well‐controlled skin manifestations, and underscores the role of IT chemotherapy in managing this complication.

## 1. Background

Primary cutaneous T‐cell lymphomas (CTCL) are hematological neoplasms that usually present only with dermatological involvement at the time of diagnosis, and the clinical course could range from indolent to a very aggressive course [1]. Recent studies have aimed to identify risk factors of central nervous system (CNS) progression in patients with peripheral T‐cell lymphoma (PTCL) [2]. However, little is known about the risk factors of CNS progression in patients with primary CTCL. Folliculotropic mycosis fungoides (FMF) is a rare variant of CTCL characterized by the infiltration of neoplastic T‐cells into the hair follicles and often has a more aggressive clinical course compared to classic MF. Earlier studies have estimated the frequency of neurological complications in CTCL to be around 3%. Half of these were due to direct CNS involvement, with leptomeningeal and parenchymal involvement accounting for most cases [3]. In this regard, we present a case of a 75‐year‐old male who presented with a CNS progression of FMF and was managed with intrathecal (IT) methotrexate.

## 2. Case Presentation

This is a case of a 75‐year‐old male who presented to the emergency department for evaluation of progressive neurologic and functional decline over the last 6 months. The patient’s medical history was notable for MF, stage IA (T1N0M0, < 10% skin involvement), which was diagnosed 4 years ago after he developed a poikilodermatous patch with scales on the left medial mandibular cheek, without palpable plaques. A skin biopsy showed dense perivascular infiltrate consisting primarily of lymphocytes and numerous eosinophils. There is a psoriasiform pattern to the epidermis, with marked spongiosis and focal exocytosis, and a significant increase in both intrafollicular and perifollicular mucin deposition. In addition, there was lymphocytic infiltration into hair follicles and destruction of associated sebaceous glands with resultant fibrosis and squamous metaplasia. These findings support the folliculotropic MF pattern of disease. PCR showed a monoclonal population of cells with the same arrangement of T‐cell receptor (TCR) gamma gene T cell. At diagnosis, peripheral blood flow cytometry and TCR clonality testing were unremarkable, consistent with B0 disease by TNMB staging. There was no evidence of blood or lymph node involvement. Initial therapy with topical steroids and bexarotene yielded little response, following which he received radiation therapy to the nose and cheek. He remained disease‐free for 3 years, after which he had disease recurrence in his left neck, which was treated with intralesional triamcinolone and radiation therapy.

He presented this admission with a progressive neurologic and functional decline including confusion, behavior changes, ataxia, fatigue, and weight loss of around 34 kg over the past 6 months. Laboratory results showed marked eosinophilia, rising from 300/μL (laboratory reference 0–600/μL) to a peak of 5400/μL, which prompted further investigation. Magnetic resonance imaging (MRI) of the brain showed T2 hyperintensity in the pons and right middle cerebellar peduncle. Cerebrospinal fluid (CSF) studies showed markedly atypical lymphocytes. PCR revealed a monoclonal T‐cell population with positive TCR gene rearrangement, and flow cytometry showed that the atypical lymphocyte population in the CSF were CD4+ and CD7−, overlapping with the immunophenotype of the patient’s MF. Given his marked eosinophilia, we tested him for myeloproliferative neoplasms, primary eosinophilia and other causes of secondary eosinophilia, all of which were negative.

The patient’s clinical presentation and neuroimaging raised concerns for CNS progression of FMF. The combination of overlap in the monoclonal T‐cell population and aberrant CD4+CD7‐immunophenotype in the CSF and skin lesion supported CNS progression of his disease. Although exceptionally rare, especially in patients with otherwise well‐controlled skin manifestations and no lymphatic involvement, the data we have are most consistent with this clinical scenario.

He was started on a high‐dose steroid taper with improvement in eosinophilia. Due to deranged liver function tests, treatment with high‐dose systemic methotrexate was deferred. The patient was started on IT chemotherapy with methotrexate and cytarabine. Significant improvement in neurological status was noted after 5 cycles of IT chemo, and CSF cytology and flow cytometry did not reveal any atypical/monoclonal lymphocyte population. The patient regained the ability to ambulate with assistance and was discharged home with a plan to continue weekly IT chemotherapy in the outpatient setting. An Ommaya reservoir was placed for subsequent administration of IT chemotherapy.

### 2.1. Investigations

• CSF study on admission: Flow cytometric coexpression of CD2 and CD4, negative for CD3, CD5, CD7, CD8, and CD57. No significant B‐cell population identified. There is a significant population of CD4+ T cell with loss of CD26 (23%). CSF also showed atypical lymphocytes, positive TCR gene arrangement, and numerous eosinophils.
• Brain MRI showed T2 hyperintensity in the pons and right middle cerebellar lobe, with low signal intensity throughout the bone marrow of the skull consistent with leptomeningeal carcinomatosis.
• Bone marrow biopsy: no evidence of involvement by lymphoma, normocellular marrow with trilineage hematopoiesis and eosinophilia; and negative TCR gene rearrangement.
• For his transaminitis, CTAP showed multiple liver cysts. Liver biopsy was without acute pathology. An ultrasound ANP vascular study showed normal abdominal vasculature with no evidence of Budd‐Chiari.
• The patient developed progression eosinophilia, rising from 300/μL (laboratory reference 0–600/μL) to a peak of 5400/μL, which prompted further investigation. Extensive workup for eosinophilia has been negative including normal vitamin B12, absence of FIP1L1‐PDGFRA mutation, lack of elevated tryptase, and negative parasitic studies.

### 2.2. Treatment

• FMF at diagnosis—topical steroids and bexarotene
• Persistent FMF—radiation therapy to the nose and cheek
• Recurrent FMF over left neck—intralesional triamcinolone followed by radiation therapy
• CNS (leptomeningeal) progression of FMF—IT methotrexate and cytarabine every 2 weeks × 6 cycles followed by every 4 weeks for 6 months.

### 2.3. Outcome and Follow‐Up

• The patient’s confusion improved after the initiation of IT chemotherapy.
• Following 2 cycles of IT chemotherapy, he became alert, oriented and reported improvement in his appetite.
• After the 5^th^ cycle, repeat CSF flow cytometry was negative for atypical monoclonal T‐cell population.
• His neurological status improved and with physical therapy, he began ambulating with support.
• He was discharged home with family support with a plan to continue IT chemotherapy in the outpatient setting.

## 3. Discussion

CTCL is a rare group of hematologic malignancies characterized by a cutaneous infiltration of malignant monoclonal T lymphocytes. MF is the most common form of CTCL, with an incidence of approximately 0.36 per 100,000 person‐years [2]. MF typically has an indolent disease course, which commonly presents as erythematous patches and plaques. The disease can progress to involve extracutaneous sites, most commonly the lymph nodes, liver, spleen, and lungs [4, 5]. CNS involvement by CTCL is exceptionally rare [6], which was found to be 1.3% and 1.6% in 2 different studies [3, 7]. FMF is a distinct variant of MF, which is characterized by the presence of folliculotropic infiltrates consisting of atypical T cells that often spare the epidermis. It has been shown to be more refractory to standard therapy and has a worse prognosis than classic MF. In the case of our patient, the overlapping phenotype of the T cells in his CSF and skin is supportive of secondary involvement of his CNS by his FMF. Although exceptionally rare, especially in patients with otherwise controlled skin manifestation and no lymphatic involvement, the data we have is most consistent with this clinical scenario.

The prognosis of CNS involvement in FMF is poor, with reported survival times in the range of 3–6 months [8, 9]. Leptomeningeal carcinomatosis originating from FMF is rarely reported in the medical literature. This is defined as an infiltration of the leptomeninges (the arachnoid membrane and the pia mater) by malignant cells, which is usually demonstrated by finding atypical cells in the CSF. Leptomeningeal carcinomatosis is associated with a poor prognosis with limited treatment options. Treatment focuses on improving quality of life and neurological symptoms and prolonging survival while minimizing toxicity. Common modalities of treatment of leptomeningeal carcinomatosis include radiation and IT administration of chemotherapy such as high‐dose methotrexate, cytarabine, and corticosteroids [10, 11].

Another interesting aspect of this case is the marked hypereosinophilia (> 5000). Eosinophilia can be classified into primary and secondary forms. Primary eosinophilia is mainly caused by clonal abnormalities of myeloid cells, while secondary eosinophilia is often reactive to the T cells’ cytokine production. Various conditions can cause secondary eosinophilia, such as parasitic infections, allergic reactions, autoimmune diseases, and malignancies [12]. Elevated eosinophil counts have been identified in malignancies; however, their association with T‐cell lymphomas has rarely been reported. Montgomery et al. identified this paraneoplastic syndrome in 0.5%–7% of cancers [13].

Hypereosinophilia develops as the malignant lymphoma cells secrete various cytokines and growth factors which stimulate the bone marrow to induce the differentiation of granulocyte precursors toward eosinophils [14]. While lymphocytic variant HES could be considered given underlying lymphoma, T‐cell clonality studies and flow cytometry make this less likely and there is rising eosinophilia from 300/μL just a few months ago to peak of 5400/μL (laboratory reference 0–600/μL) appears more consistent with a paraneoplastic phenomenon. Our patient had an extensive negative workup including normal vitamin B12, absence of FIP1L1‐PDGFRA mutation, lack of elevated tryptase, and negative parasitic studies, which helps exclude myeloproliferative variant HES and other secondary causes. This would be mostly suggestive of CNS lymphoma with paraneoplastic eosinophilia as a unifying diagnosis. The present clinical case brings forward a complex diagnostic scenario of great importance for medical oncologists and hematologists.

## Consent

All the patients allowed personal data processing and informed consent was obtained from all individual participants included in the study.

## Conflicts of Interest

The authors declare no conflicts of interest.

## Author Contributions

Cher Ying Foo: conceptualization, writing–original draft, and writing–review and editing.

Aditya Sanjeevi: conceptualization and writing–original draft.

Harkarandeep Singh: supervision and review.

## Funding

No funding was received for this manuscript.

## Data Availability

The data that support the findings of this study are available from the corresponding author upon reasonable request.
